# Effect of Ultrasonic Irradiation on the Physicochemical and Structural Properties of *Laminaria japonica* Polysaccharides and Their Performance in Biological Activities

**DOI:** 10.3390/molecules28010008

**Published:** 2022-12-20

**Authors:** Jinhui Wu, Huiying Wang, Yanfei Liu, Baojun Xu, Bin Du, Yuedong Yang

**Affiliations:** 1 Hebei Key Laboratory of Natural Products Activity Components and Function, Hebei Normal University of Science and Technology, Qinhuangdao 066004, China; 2 Food Science and Technology Program, Department of Life Sciences, BNU–HKBU United International College, Zhuhai 519087, China

**Keywords:** *Laminaria japonica* polysaccharide, ultrasound, physicochemical characteristics, anti-inflammatory, RAW264.7 macrophages, antioxidant activity

## Abstract

Due to the large molecular weight and complex structure of *Laminaria japonica* polysaccharides (LJP), which limit their absorption and utilization by the body, methods to effectively degrade polysaccharides had received more and more attention. In the present research, hot water extraction coupled with three-phase partitioning (TPP) was developed to extract and isolate LJP. Ultrasonic *L. japonica* polysaccharides (ULJP) were obtained by ultrasonic degradation. In addition, their physicochemical characteristics and in vitro biological activities were investigated. Results indicated that ULJP had lower weight-average molecular weight (153 kDa) and looser surface morphology than the LJP. The primary structures of LJP and ULJP were basically unchanged, both contained α-hexo-pyranoses and were mainly connected by 1,4-glycosidic bonds. Compared with LJP, ULJP had stronger antioxidant activity, α-amylase inhibitory effect and anti-inflammatory effect on RAW264.7 macrophages. The scavenging rate of DPPH free radicals by ULJP is 35.85%. Therefore, ultrasonic degradation could effectively degrade LJP and significantly improve the biological activity of LJP, which provided a theoretical basis for the in-depth utilization and research and development of *L. japonica* in the fields of medicine and food.

## 1. Introduction

Named for its scallop shape, *Laminaria japonica* is a widely consumed medicinal and edible brown algae. *L. japonica* is cultured in a large area in the coastal areas of China and the annual output is also increasing year by year, so the cost is very low [1,2]. *L. japonica* has high nutritional value, not only rich in vitamins and minerals, but also containing polysaccharides, crude protein, crude fiber and so on [3,4,5]. Therefore, *L. japonica* can promote food digestion, beauty, treatment of constipation and other functions [6]. *L. japonica* also has health care functions, such as regulating blood sugar and blood lipids, enhancing human resistance, lowering blood pressure, etc. [7]. In addition, there are still many effects of *L. japonica* that we need to study and develop and the market has great potential for development.

*Laminaria japonica* polysaccharides (LJP) is one of the main active components of *L. japonica* and is a sulfated polysaccharide that exhibits various biological activities and health benefits, such as anti-inflammatory, antioxidant, hypoglycemic, immunomodulatory and antibacterial effects [8,9,10,11,12,13,14]. In recent years, many bioactive polysaccharides have been extracted from dried *L. japonica* samples by physical, chemical and biological methods [15,16,17]. However, natural polysaccharides have high molecular weight, complex structure, high viscosity, difficult diffusion and are difficult to be absorbed by the body and natural high molecular polysaccharides cannot pass through various barriers of the body and even cell membranes. Changes in polysaccharide molecular weight can lead to changes in polysaccharide structure and biological activity [18]. Therefore, it is necessary to develop a treatment method that can not only maintain the biological activity of LJP, but also reduce its molecular weight, so as to improve the utilization rate of LJP. Moreover, Bhadja et al. [19] investigated six low-molecular-weight seaweed polysaccharides (SPSs) on oxalate-induced damaged human kidney proximal tubular epithelial cells (HK-2). The results showed that these SPSs within 0.1–100 μg/mL did not express cytotoxicity in HK-2 cells and each polysaccharide had a repair effect on oxalate-induced damaged HK-2 cells.

At present, there are various degradation methods for polysaccharides, including acid, oxidative, enzymatic, ultrasonic degradation [20,21,22,23,24,25] and ultraviolet/hydrogen peroxide method [26]. Among them, the ultrasonic degradation method has the advantages of low cost, high efficiency, simple operation and less side reactions, which is favorable for wide-scale popularization and use. It is speculated that the ultrasonic degradation method can degrade the polysaccharide well and maintain biological activity. However, to the best of our knowledge, there are few studies on reducing the molecular weight of LJP to improve bioactivity based on ultrasonic degradation of LJP.

Therefore, in this study, ULJP were obtained by ultrasonic degradation and the physicochemical properties, structural characteristics and in vitro biological activities of LJP and ULJP were studied and compared. This study provides a theoretical basis for the in-depth utilization and research and development of *L. japonica* in the fields of medicine and food.

## 2. Results and Discussion

### 2.1. Physicochemical Properties of LJP and ULJP

The chemical analysis results are shown in Table 1. The molecular weights of LJP and ULJP are one of the important parameters affecting their biological activities, such as anti-inflammatory and antioxidant properties. As shown in Table 1, the Mw, Mn and Mp of ULJP are significantly lower than those of LJP, which indicates that ultrasonic degradation of LJP is effective, The degraded ULJP is a low molecular weight polysaccharide; the larger the polydispersity index, the wider the molecular weight distribution, and the polydispersity index of ULJP is significantly higher than that of LJP, which indicates that ultrasonic degradation effectively degrades high molecular weight LJP into a variety of low molecular weight polysaccharides; too many low molecular weight polysaccharides may cause the polydispersity index of ULJP to be significantly higher than that of LJP [27]. Mao et al. [28] examined the impact of power ultrasound on the molecular properties of a high-molecular weight exopolysaccharide from the Cs-HK1 medicinal fungus. The results showed that the area percentage or the relative proportion of the higher Mw peak in the order of 10^7^ decreased, while that of lower Mw of 10^6^ decreased with the ultrasound treatment period. In another study, Chen et al. [26] produced the polysaccharides from *Laminaria japonica* by the treatment of ultraviolet/hydrogen peroxide (UV/H2O2) degradation. The molecular weight of LJP decreased from 315 kDa to 20 kDa. The reason for this finding is probably the different degradation method [26]. 

The monosaccharide composition of LJP and ULJP was analyzed by HPAEC and Table 1 shows the results of monosaccharide composition content of LJP and ULJP. Compared with LJP, the content of fucose, mannose and uronic acid in ULJP decreased significantly, the content of fucose decreased from 13.21% to 0.93%, the content of mannose decreased from 4.97% to 0.56% and the content of uronic acid decreased from 68.42% to 5.37%. Compared with LJP, the galactose content of ULJP increased significantly, from 9.37% to 72.56%, presumably because the fucoidan in LJP was degraded into galactose by ultrasonic. Compared with LJP, the glucose content of ULJP increased significantly, from 0.61% to 16.87%, presumably because the fucoidan in LJP was degraded into glucose ultrasonically [29]. In conclusion, sonication is a practical method to degrade polysaccharides with high efficiency.

### 2.2. SEM Picture Analysis of LJP and ULJP

**Figure 1:**
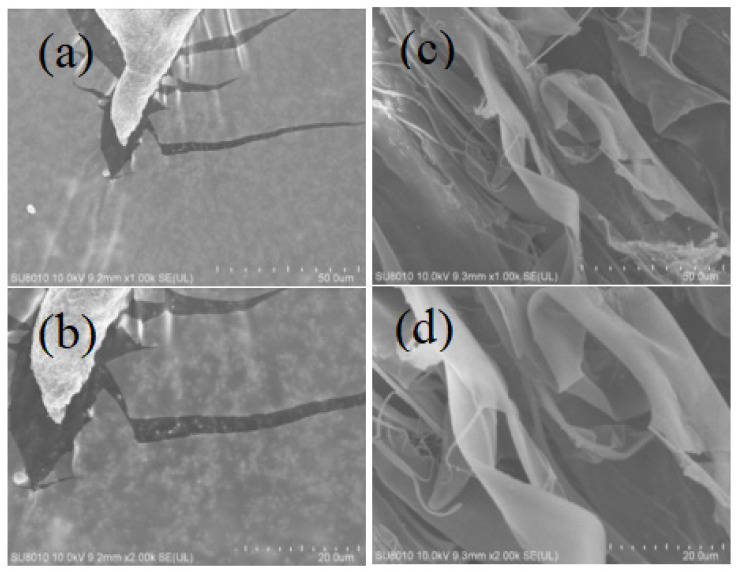
SEM picture of LJP and ULJP. (**a**) ×1.00 k and (**b**) ×2.00 k LJP; (**c**) ×1.00 k and (**d**) ×2.00 k ULJP.

As can be seen from Figure 1a,b, the surface morphology of LJP is smooth and flaky; from Figure 1c,d, it can be seen that ULJP is loosely dispersed in fibrous piles, indicating that LJP is degraded into ULJP of various small molecules ultrasonically [30]. This ULJP looks like thin film with a smooth and glittering surface. Furthermore, the SEM scan showed that the exopolysaccharide was made of a homogeneous matrix.

### 2.3. FT-IR Analysis of LJP and ULJP

**Figure 2:**
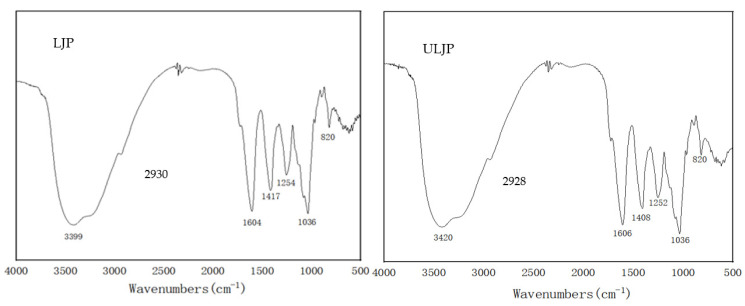
IR spectrum of LJP and ULJP.

Infrared spectroscopy is often used to identify the characteristic functional groups of polysaccharides. Figure 2 shows the infrared spectral results of LJP and ULJP, respectively. The characteristic peaks of polysaccharides are O-H and C-H stretching vibration peaks at 3400 and 2900 cm^−1^ and the characteristic absorption peak of C=O asymmetric stretching vibration is 1600 cm^−1^, indicating that LJP and ULJP contain uronic acid; 1417 cm^−1^ may be caused by the bending vibration of C-H; the characteristic absorption peak of the stretching vibration of the C-O-C bond of pyranoside is a narrow and strong absorption peak around 1000 cm^−1^. The stretching vibration peak of O=S=O asymmetry in sulfate is a strong and broad absorption peak at 1250 cm^−1^, and the characteristic absorption peak of C-O-S stretching vibration and the characteristic peak of sulfate bond are absorption peaks at 820 cm^−1^, indicating that LJP and ULJP do contain sulfate groups. The functional groups of LJP and ULJP are almost the same, indicating that ultrasonic degradation hardly changes the functional groups of polysaccharides and does not change their primary structure [31]. The important information of the characteristic functional groups in the polysaccharide structure can be qualitatively obtained by the FT-IR map results, but the structural differences between LJP and ULJP cannot be quantitatively analyzed.

### 2.4. NMR Spectra Analysis of LJP and ULJP

**Figure 3:**
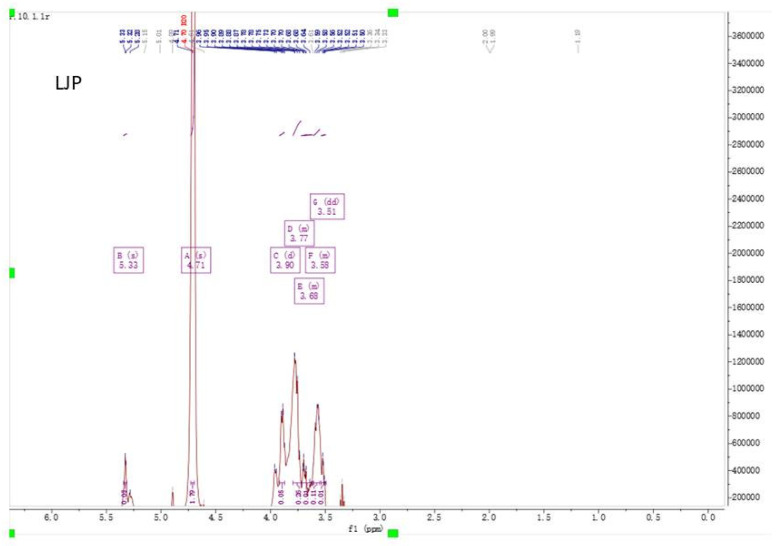

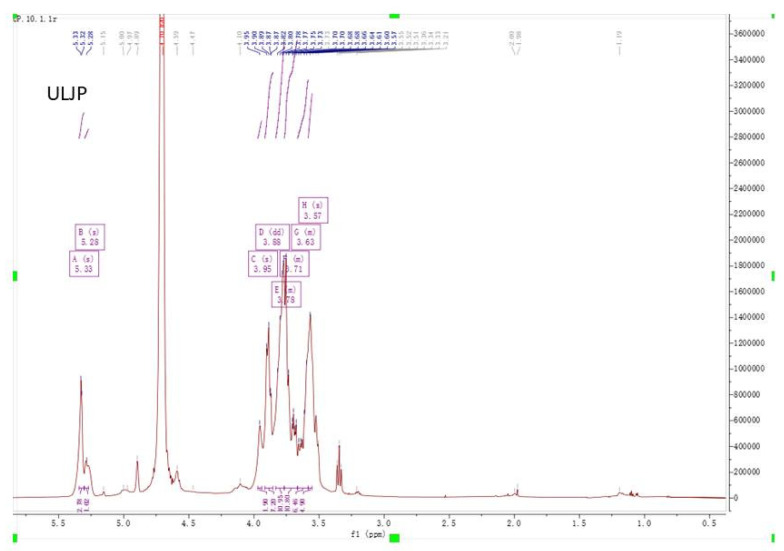
NMR spectra of LJP and ULJP (solvent = D_2_O): ^1^H NMR spectrum.

The shielding effect of the hydroxyl group results in the chemical shift of H on the sugar ring carbon, located at 3–4 ppm, which is difficult to resolve. However, the chemical shift of the anomeric hydrogen is in the lower field, between 4.5 and 5.5 ppm, and the monosaccharide species has the same number of proton signals in this region. More than 4.95 ppm is the chemical shift of α-pyranose H and less than 4.95 ppm is the chemical shift of β-pyranose H, so as to determine the sugar ring configuration. In order to further verify the structural information of LJP and ULJP, 1H-NMR spectrum analysis was carried out, as shown in Figure 3, the ^1^H-NMR spectrum of LJP has a total of 7 peaks, 1H NMR (600 MHz, Deuterium Oxide) δ 5.33 (s, 0H), 4.71 (s, 2H), 3.90 (d, J = 7.9 Hz, 0H), 3.79~3.72 (m, 0H), 3.72~3.61 (m, 0H), 3.61~3.55 (m, 0H), 3.51 (dd, J = 9.4, 3.6 Hz, 0H); as shown in Figure 3, the ^1^H-NMR spectrum of ULJP has 8 peaks, ^1^H NMR (600 MHz, Deuterium Oxide) δ 5.33 (s, 3H), 5.28 (s, 1H), 3.95 (s, 2H), 3.88 (dd, J = 14.2, 5.5 Hz, 7H), 3.83~3.77 (m, 11H), 3.77~3.66 (m, 11H), 3.66~3.58 (m, 6H), 3.57 (s, 5H). The ^1^H-NMR of LJP has an anomeric hydrogen signal, δ 5.33 (s, OH) and the chemical shift is greater than 4.95 ppm, indicating that it is α-pyranose, that is, LJP contains glucose and the chemical shift peak integral area is small, corresponding to the single sugar composition, and the glucose content is low, only 0.61%; the ^1^H-NMR of LJP also has an anomeric hydrogen signal, δ 5.33 (s, 3H) and the chemical shift is greater than 4.95 ppm, indicating that it is α-pyranose, that is, ULJP contains glucose, the chemical shift peak integral area is larger, at 3H, corresponding to the monosaccharide composition and the glucose content is relatively high, at 16.87% [32].

**Figure 4:**
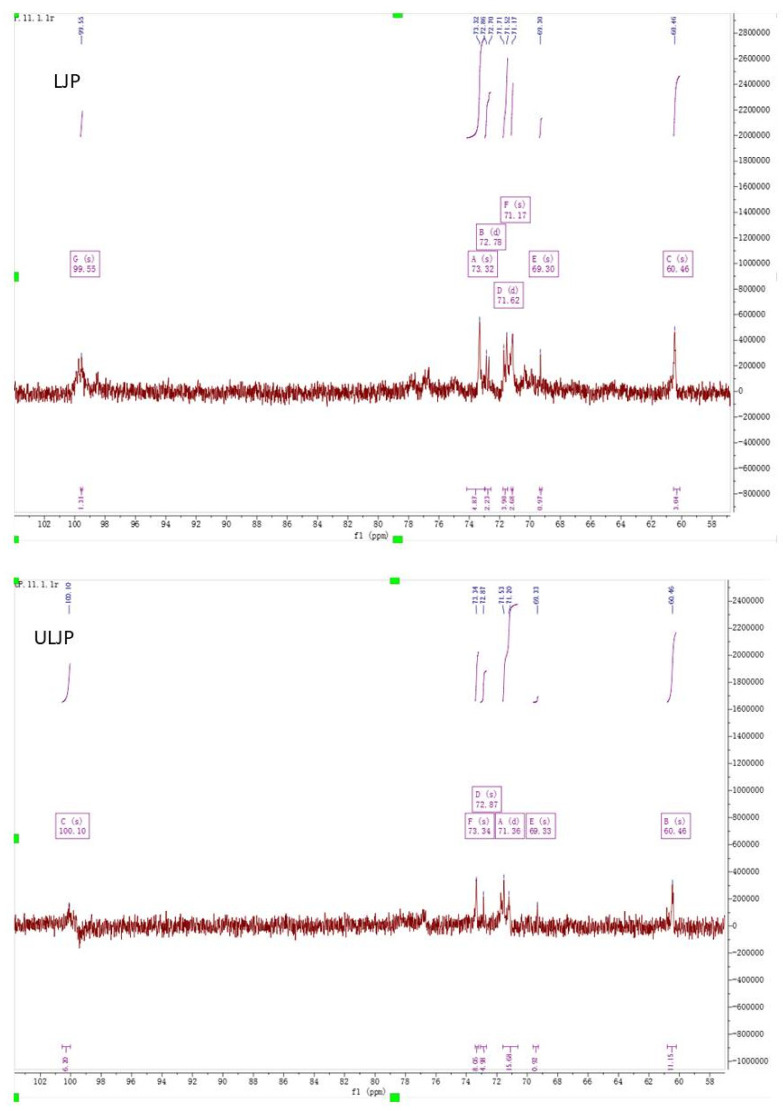
NMR spectra of LJP and ULJP (solvent = D_2_O): ^13^C NMR spectrum.

The chemical shift range of ^13^C-NMR is wider than that of ^1^H-NMR, up to 300 ppm, the anomeric carbon of polysaccharides is between 695 and 110 ppm and the composition of monosaccharides is the same as the number of signals in this range. The conformation of the substituent has a great influence on the chemical shift. For example, the substituent on the anomeric carbon is a vertical bond in the high field. According to this, the sugar ring configuration, D-glucose, α-δ is 97–101 ppm and β-δ is 103 ppm~106 ppm. In order to further verify the structural information of LJP and ULJP, ^13^C-NMR spectrum analysis was carried out. As shown in Figure 4, the ^13^C-NMR spectrum of LJP has a total of 8 peaks, ^13^C NMR (151 MHz, Deuterium Oxide) δ 99.55, 78.86, 73.32, 72.78 (d, J = 24.8 Hz), 71.62 (d, J = 28.9 Hz), 71.17, 69.30, 60.46. As shown in Figure 4, there are 7 peaks in the ^13^C-NMR spectrum of ULJP, ^13^C NMR (151 MHz, Deuterium Oxide) δ 100.10, 78.86, 73.34, 72.87, 71.36 (d, J = 50.1 Hz), 69.33, 60.46. The 13C-NMR of LJP has an anomeric carbon signal, δ 99.55, indicating that it consists of a monosaccharide, which is α-glucose; the ^13^C-NMR of ULJP also has an anomeric carbon signal, δ 100.10, indicating that it consists of a monosaccharide, α-glucose, which is the same as the ^1^H-NMR result. LJP ^13^C-NMR 60.46 ppm is the chemical shift of unsubstituted C6; 78.86, 73.32, 72.78, 71.62, 71.17, 69.30 is the signal of non-terminal carbon (C2, C3, C4 and C5), the peak of substituted proton signal being 78.86 ppm. This indicates that LJP is 1,4-glycosidic linkage. ULJP ^13^C-NMR 60.46 ppm is the chemical shift of unsubstituted C6, 73.34, 72.87, 71.36, 69.33 are non-terminal carbon (C2, C3, C4 and C5) signals, and the peak of substituted proton signal 78.86 ppm indicates that LJP is 1,4-glycosidically linked [33].

Comprehensive analysis of ^1^H-NMR and ^13^C-NMR spectral results confirmed that LJP and ULJP contain α-hexo-pyranoses, which are mainly connected by 1,4-glycosidic bonds. Ultrasonic degradation can effectively degrade the fucoidan of macromolecular LJP into small molecule ULJP, such as glucose.

### 2.5. Antioxidant Activity of LJP and ULJP In Vitro

**Figure 5:**
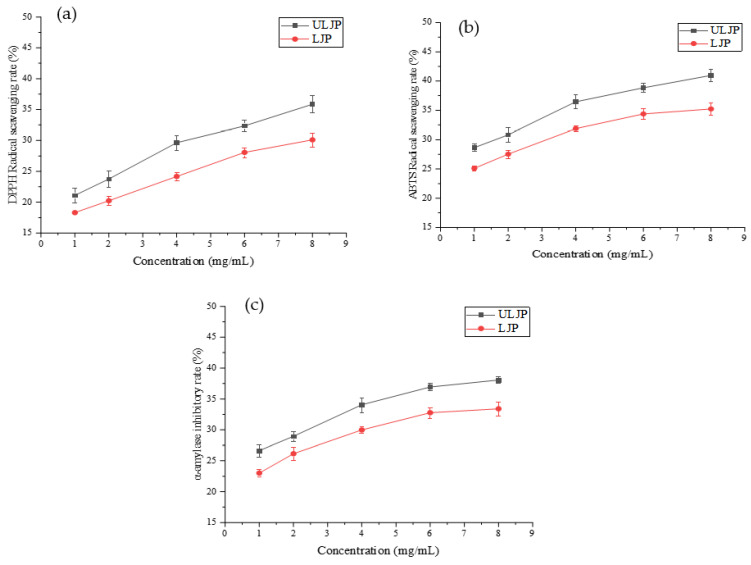
(**a**) Scavenging rate of DPPH free radical by ULJP and LJP. (**b**) Scavenging rate of ABTS free radical by ULJP and LJP. (**c**) Inhibitory rate of α-amylase by ULJP and LJP.

It can be seen from Figure 5a that the scavenging rate of DPPH free radicals by ULJP and LJP is proportional to the concentration. At 8 mg/mL, the scavenging rate of ULJP is 35.85% and the scavenging rate of LJP is 30.11%. Compared with LJP, the scavenging rate of ULJP is increased by 16.01%. Therefore, the antioxidant activity of ULJP is stronger than that of LJP. This result is similar to that of Du et al. [34]. The corncob polysaccharide was extracted by cellulase enzymatic hydrolysis and its DPPH free radical scavenging rate was significantly stronger than that of the corncob polysaccharide before enzymatic hydrolysis. The molecular weight is reduced and it is easier to contact and react with DPPH free radicals, thereby improving the antioxidant activity of degrading polysaccharides.

It can be seen from Figure 5b that the scavenging rate of ULJP and LJP to ABTS free radicals is proportional to the concentration. At 8 mg/mL, the scavenging rate of ULJP is 40.97% and the scavenging rate of LJP is 35.22%. Compared with LJP, the scavenging rate of ULJP is increased by 14.03%. Therefore, the antioxidant activity of ULJP is stronger than that of LJP. This is in agreement with many previously published results which showed that, using enzyme and a slight acid degradation method to degrade astragalus polysaccharide, its ABTS free radical scavenging rate is significantly stronger than that of polysaccharide before degradation and its small molecular weight (<3 kDa, 3–10 kDa and >10 kDa) degraded astragalus polysaccharides have good antioxidant capacity in vitro. This is because the degraded small molecular weight polysaccharides have loose structure and are easy to contact and react with ABTS free radicals. Therefore, astragalus polysaccharides degraded by enzymes and slight acid degradation methods also reflect better antioxidant activity [35].

### 2.6. Hypoglycemic Activity of LJP and ULJP In Vitro

As can be seen from Figure 5c, the inhibition rates of ULJP and LJP on α-amylase increased significantly with the increase of the corresponding concentration. Compared with LJP, the inhibition rate of ULJP on α-amylase increased by 12.27%. Therefore, the inhibition rate of ULJP on α-amylase is greater than that of LJP, that is, the hypoglycemic activity of ULJP is stronger than that of LJP. There were some other reports presenting similar phenomena to those of this work. The H_2_O_2_-Fe^2+^ methods were used to degrade the pine algae polysaccharide, the solubility of the pine algae polysaccharide after degradation was significantly improved, the particle size was reduced and the inhibitory activity to α-amylase was significant. The hypoglycemic activity of degraded polysaccharides was also significantly enhanced [36]. It has been reported that the hypoglycemic activity of polysaccharide is not only related to the Mw, but also to monosaccharide composition. Yang et al. [37] investigated that a low molecular weight heteropolysaccharide from the fruiting body of *Phellinus pini* exhibitedα-glucosidase inhibition and glucose consumption amelioration in an insulin-resistant HepG2 cell model. The Mw of this polysaccharide is 3.2 kDa.

### 2.7. Effect of LJP and ULJP on RAW264.7 Cell Viability

**Figure 6:**
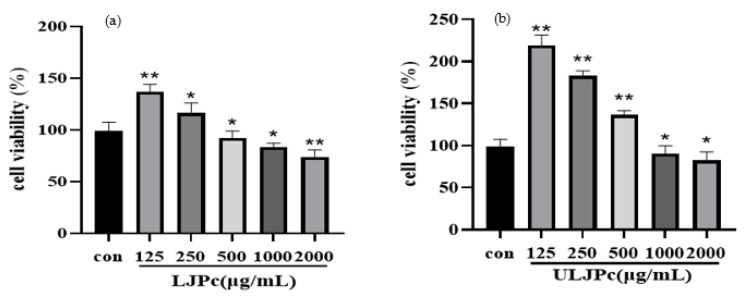
(**a**) Effects of LJP on the proliferation and viability of RAW264.7 macrophages; (**b**) Effects of ULJP on the proliferation and viability of RAW264.7 macrophages. (* *p* < 0.05; ** *p* < 0.01).

The effects of LJP and ULJP on the proliferation activity of RAW264.7 macrophages were detected by CCK-8 method and the results are shown in Figure 6a,b. The proliferation activity of RAW264.7 macrophages was negatively correlated with the concentrations of LJP and ULJP, respectively. When the LJP concentration was 500 μg/mL, the proliferation activity of RAW264.7 macrophages was 92.08%, lower than 100%. At this time, LJP showed an inhibitory effect on cell proliferation; when the concentration of ULJP was 1000 μg/mL, and the proliferation activity of RAW264.7 macrophages was 90.45%, which was lower than 100%. At this time, ULJP showed an inhibitory effect on cell proliferation. Therefore, according to the test results and the actual test, the cytotoxicity of the sample itself should be excluded. For the subsequent activity test of RAW264.7 macrophages, 100, 200, 300 µg/mL can be selected as the test concentrations. This is consistent with the study of Wu [38]; when the concentration of LJP is less than 500 µg/mL, it has no inhibitory effect on the proliferation of RAW264.7 macrophages.

### 2.8. Effect of LJP and ULJP on the RAW264.7 Macrophages

**Figure 7:**
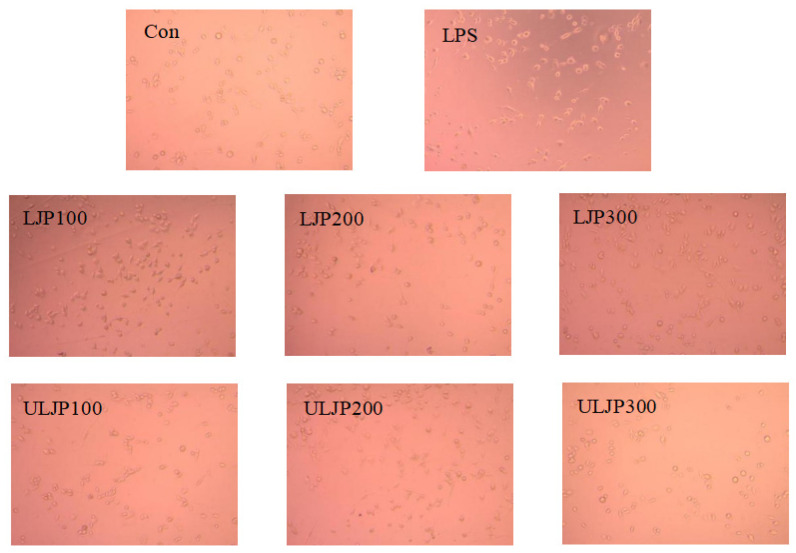
Effects of LJP and ULJP on the morphology of RAW264.7 macrophages.

RAW264.7 macrophages usually showed three morphologies: round, slender and obvious branched extensions. The optimal experimental conditions were used for LPS incubation time of 16 h, concentration of 1 µg/mL and incubation time of LJP and ULJP for 2 h to explore the effects of LJP and ULJP on the morphology of RAW264.7 macrophages. As shown in Figure 7, the morphology of RAW264.7 macrophages in the Con control group was round or oval, which was consistent with the normal shape. The morphology changed, the cell volume increased significantly and some antennae were visible around the cells. When LJP and ULJP were added to the DMEM medium that had been added with LPS, compared with the Con control group, the cell body of RAW264.7 cells was gradually enlarged, the antennae were branched and the number of antennae increased significantly. The size of the RAW264.7 macrophages in the LPS group was not as large as that in the LPS group. This morphology could increase the contact area with external substances, which was beneficial to the adherence ability of the RAW264.7 macrophages. When the concentration of LJP and ULJP increased, the number of antennae of RAW264.7 macrophages decreased and the volume was also relatively reduced, which indicated that both LJP and ULJP had the ability to inhibit the antennae of RAW264.7 macrophages; and at the same concentration of ULJP, the inhibitory effect of the experimental group was better than that of the LJP experimental group.

### 2.9. Effect of LJP and ULJP on RAW264.7 Phagocytic Activity

**Figure 8:**
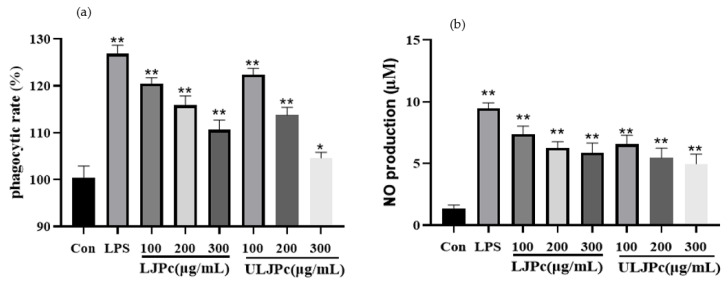
Effects of LJP and ULJP on the morphology of RAW264.7 macrophages. (* *p* < 0.05; ** *p* < 0.01). (**a**) phagocytic rate (%) (**b**) NO production µM.

Activated macrophages help initiate specific defense mechanisms and play a key role in the immune system and phagocytosis is a key indicator of macrophage activity. The phagocytic activity of macrophages was determined by the neutral red uptake assay. From Figure 8a, the phagocytosis of RAW264.7 macrophages by LJP and ULJP decreased in a concentration-dependent manner and, at the same concentration, the inhibitory effect of LJP on the phagocytic activity of RAW264.7 macrophages was lower than that of ULJP. The results showed that both LJP and ULJP could inhibit the phagocytosis of RAW264.7 macrophages and at the same concentration, ULJP had a better inhibitory effect on the phagocytosis of RAW264.7 macrophages than LJP, which was consistent with the morphological characteristics of the cells.

### 2.10. Effect of LJP and ULJP on RAW264.7 NO Production

When RAW264.7 macrophages are stimulated, they can alternately be activated to pro-inflammatory subtypes (M1 macrophages) or anti-inflammatory and tissue repair subtypes (M2 macrophages). M1 and M2 cells have opposite effects, M1 macrophages phages have pro-inflammatory effects and release NO and other pro-inflammatory factors, while M2 macrophages have immunosuppressive effects. NO is an indispensable bioactive molecule in the body. As shown in Figure 8b, compared with the LPS group, the NO release of LJP and ULJP was always lower than that of the LPS group, indicating that LJP and ULJP can reduce the release of NO from macrophages. It has an anti-inflammatory effect on macrophages. Adding different concentrations of LJP and ULJP will reduce the release of NO from RAW264.7 macrophages. When the concentration of LJP is 300 µg/mL, the release of NO reaches 5.87 µmol/L (*p* < 0.01), which was 0.74 times that of the control group. When the ULJP concentration was 300 µg/mL, the release of NO reached 4.97 µmol/L (*p* < 0.01), indicating that at the same concentration of ULJP, the anti-inflammatory activity is better than that of LJP.

In this work, LJP and ULJP can reduce the release of NO from cells. LJP and ULJP stimulate RAW macrophages to differentiate into M2 macrophages, thereby reducing NO release. Therefore, it is speculated that LJP can improve non-specific immune function of ULJP. The reduction of NO release in RAW macrophages was significantly lower than that in LJP, indicating that ultrasonic degradation treatment can improve the anti-inflammatory activity of ULJP, which may be due to the smaller molecular weight and the active groups contained in ULJP, such as sulfate groups, are more exposed. It is easier to combine with cells, so it is easier to show strong anti-inflammatory activity.

### 2.11. Effects of LJP and ULJP on mRNA Expression Levels of Inflammatory Factors in LPS-Induced RAW264.7 Macrophages

**Figure 9:**
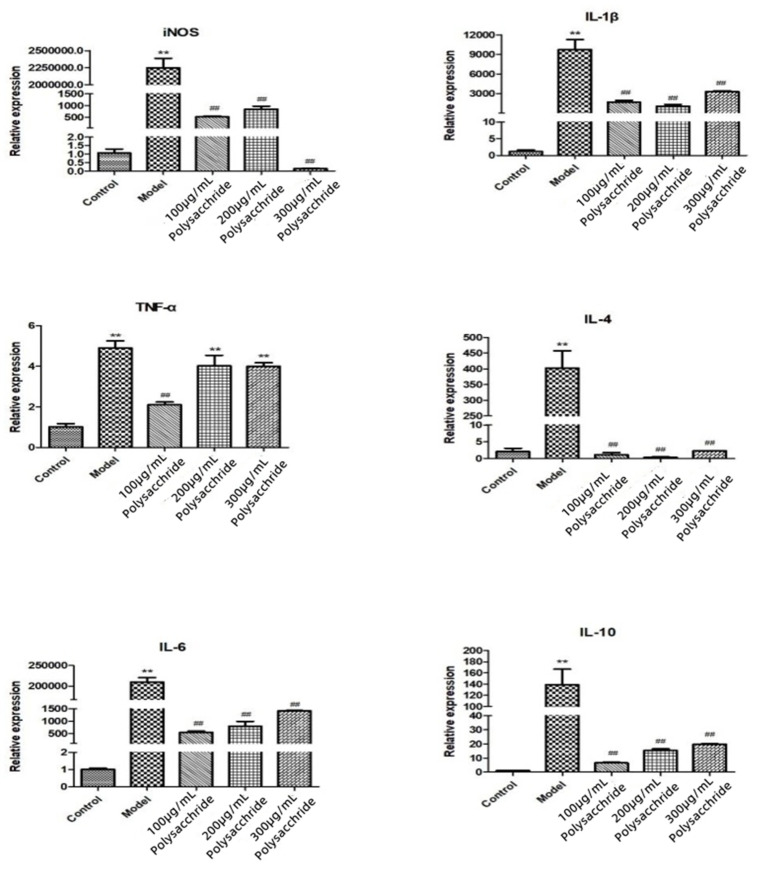
Effects of LJP on mRNA expression levels of inflammatory factors in LPS-induced RAW264.7 macrophages. (## *p* < 0.05; ** *p* < 0.01).

**Figure 10:**
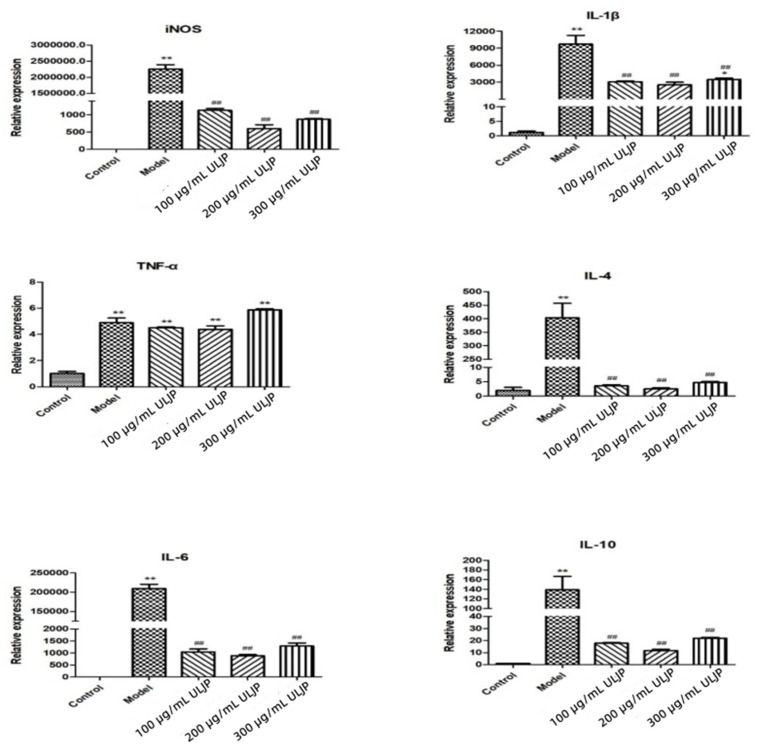
Effects of ULJP on mRNA expression levels of inflammatory factors in LPS-induced RAW264.7 macrophages. (## *p* < 0.05; ** *p* < 0.01; * *p* < 0.05).

The effects of LJP and ULJP on the expression levels of LPS-induced RAW264.7 macrophages are shown in Figure 9 and Figure 10. The results showed that the mRNA expression levels of IL-4, iNOS, IL-1β, IL-6, IL-10, TNF-α and IFN-γ in macrophages were significantly increased with the addition of LPS. The mRNA expression of each inflammatory factor showed a downward trend as a whole. Under the same inflammatory factor and concentration, the inhibitory effect of ULJP was significantly better than that of LJP and the difference was significant compared with the LPS group (*p* < 0.01). That is, ULJP treated by ultrasonic degradation has more excellent anti-inflammatory activity. This result is the same as that of Qiu et al. [39]. Treatment of RAW264.7 macrophages with total flavonoids of gang pine promoted cell proliferation (*p* < 0.05) and inhibited the contents of IL-1β, IL-8 and TGF-β (*p* < 0.05), up-regulated the relative expression of IκBα protein (*p* < 0.05), inhibited the mRNA gene expression (mRNA) of NF-κB p65, and the mRNA expression level of NF-κB p65 was concentration-dependent with total flavonoids It is speculated that the inhibition of NF-κB signaling pathway by total flavonoids of pineapple may be one of the mechanisms of its anti-inflammatory activity.

## 3. Materials and Methods

### 3.1. Materials and Chemicals

Fresh raw *Laminaria japonica* (Place of origin: Qinhuangdao, China), purchased from a local supermarket (Qinhuangdao, Hebei, China) in April 2019, were free from pests and mechanical damage. All chemicals and reagents were of laboratory grade and provided by local authorized suppliers.

### 3.2. Extraction and Isolation of LJP and ULJP

Hot water extraction coupled with three-phase partitioning (TPP) was developed to extract and isolate LJP from *L. japonica*. According to Wang et al. [40], with some modifications, in short, the optimal extraction conditions for LJP are extraction temperature 70 °C, extraction time 2 h, water-material ratio 50 mL/g, extraction times 2. According to Yan et al. [41], with some modifications, TPP was used to isolate LJP. Briefly, 20 g of *L. Japonica* powder, add 800 mL of distilled water were stirred for 2 h at 70 °C and then subjected to centrifugation (7000× *g*, 5 min) for supernatant collection. Then, 20% (*w*/*w*) (NH_4_)_2_SO_4_ was added to the resulting supernatant and stirred until completely dissolved. Subsequently, the mixture was mixed with 1.5 vol oft-butanol (*v*/*v*) and continuously stirred at 35 °C for 30 min. After that, the resultant mixture was centrifuged at 4000× *g* for 15 min, the collected lower aqueous phase was subjected to dialysis (MWCO: 3500 Da) in distilled water for 48 h and then concentrated to half.

### 3.3. Ultrasonic Degradation of LJP to Prepare ULJP

Referring to Wu et al. [42], with a slight modification, the LJP after dialysis and concentration was degraded by a Scientz-IID ultrasonic cell disrupter (Ningbo Scientz Biotechnology Company, Ltd., Ningbo, Zhejiang, China) for 5 h. The frequency is 25 kHz and maximum output power is 950 W. Each ultrasonic wave was 1 h, stopped for 10 min when water was replaced and, after ultrasonic degradation, freeze-dried to obtain ultrasonic *L. japonica* polysaccharide (ULJP).

### 3.4. Determination of the Physicochemical Characteristics of LJP and ULJP

#### 3.4.1. General Analysis

The total sugar, protein, uronic acid and sulfate group contents of polysaccharide were separately determined with the phenol-sulfuric acid, sulfuric acid-carbazole, Bradford and Barium sulfate-turbidimetry methods [43,44,45,46].

#### 3.4.2. Determination of Molecular Weight and Polydispersity

According to the method of Chen et al. [47] to determine the molecular weight by gel permeation chromatography (GPC), with a slight modification, the molecular weight of LJP and ULJP was determined by GPC. Polysaccharide was characterized for molecular weight using Agilent 1100 series HPLC system (Agilent Technologies Palo AHO, CA, USA) equipped with a TOSOH TSK-GEL G3000 SW XL column (7.8 mm × 30 cm, 10 µm; TOSOH Corp., Tokyo, Japan) and an refractive index detector. A sample of 20 µL was injected in the system by maintaining a flow rate of 0.5 mL min^−1^ and column temperature of 35 °C. Separation was carried out using 0.05 mol L^−1^ phosphate buffer (pH 6.7) containing 0.05% NaN_3_ as mobile phase. The average molecular weight was calculated by the calibration curve obtained using various standard dextrans (738, 5800, 11,220, 21,370, 41,800, 110,000, 118,600, 318,000 and 813,500).

#### 3.4.3. Monosaccharide Composition Analysis

Refer to Li et al. [48] for the determination of monosaccharide composition in polysaccharides by acid hydrolysis and high performance anion exchange chromatography (HPAEC), with minor modifications. LJP (5 ± 0.05 mg) was put into a tube and 0.5 mL of 12 M H_2_SO_4_ was added in an ice bath with magnetic stirring for 0.5 h to dissolve it completely. After 2 mL of ultrapure water was added, tubes were placed in an oil bath at 105 °C for 4 h. After heating, polysaccharide solution was fixed the volume with a 250 mL volume volumetric flask, 1 mL of sample was taken out and was filtered through 0.22 µm membrane for HPAEC analysis. Based on the use of a pulsed ampere-metric detector (PAD; Dionex ICS 5000 system) on a CarboPac PA-20 anion exchange column (3 × 150 mm; Dionex) on the monosaccharide composition content of LJP and ULJP were analyzed by high performance anion exchange chromatography (HPAEC). Flow rate, 0.5 mL/min; injection volume, 5 μL; solvent system, B: (0.1 M NaOH, 0.2 M NaAc); gradient program, 95:5 *v*/*v* at 0 min, 80:20 *v*/*v* at 30 min, 60:40 *v*/*v* at 30.1 min, 60:40 *v*/*v* at 45 min, 95:5 *v*/*v* at 45.1 min and 95:5 *v*/*v* at 60 min.

#### 3.4.4. Scanning Electron Microscopy Analysis

The morphology of LJP and ULJP was observed by scanning electron microscopy [49]. Samples were fixed on aluminum stub and gold sputtered and examined through KYKY-2800 SEM (KYKY Technology Co., Ltd., Beijing, China) by maintaining an accelerated voltage of 10 kV

#### 3.4.5. Fourier Transform Infrared Analysis

Fourier transform infrared (FT-IR) spectra of freeze-dried LJP and ULJP were captured on a Nexus 670 FT-IR spectrometer in the wavenumber range of 4000–500 cm^−1^ with KBr pellets referenced against air, according to the method by Kibar et al. [50].

#### 3.4.6. NMR Spectra Analysis

According to the method of Takis et al. [51] to measure the sample by nuclear magnetic resonance spectrometer, with some modifications, LJP and ULJP were dissolved in D_2_O respectively and Bruker AVANCE III HD 600 MHz NMR was used.

### 3.5. Antioxidant Activity Evaluation of LJP and ULJP In Vitro

DPPH radical scavenging capacity of samples was evaluated according to the method of Insang et al. [52,53] with slight modifications. Two mL of sample extract and 2.0 mL of 0.1 mmol/L DPPH in ethanol were briefly mixed in a test tube. Before standing in darkness for 30 min at 37 °C, the mixture solution was mixed for 1 min with a vortex. Vitamin C was used as the positive control and ethanol was used as blank. The absorbance was measured at 517 nm with a visible light spectrophotometer. The experiment was carried out in triplicate. Radical scavenging activity was calculated using the following formula:

DPPH inhibition = [1 − (A_1_ − A_2_)/A_0_] × 100% (1)

where A_0_ = absorbance of 2 mL DPPH-ethanol + 2 mL ethanol; A_1_ = absorbance of 2 mL DPPH-ethanol + 2 mL sample; A_2_ = absorbance of 2 mL ethanol + 2 mL sample.

ABTS radical cation (ABTS+•) was produced by reacting ABTS solution (7.4 mM) with 2.6 mM K_2_S_2_O_8_ and allowing the mixture to stand in the dark at room temperature for 12–16 h before use. For the study, the ABTS+• solution was diluted in deionized water or ethanol to an absorbance of 0.7 (±0.02) at 734 nm. An appropriate solvent blank reading was taken. After the addition of 100 μL of sample solutions to 3 mL of ABTS+• solution, the absorbance reading was taken at 30 °C, 10 min after initial mixing. All solution was used on the day of preparation and all determinations were carried out in triplicate [54]. The percentage of inhibition of ABTS+ was calculated using the following formula:

ABTS^+•^inhibition = [1 − (A_1_ − A_2_)/A_0_] × 100% (2)

where A_0_ = absorbance of ABTS + dilute solution + water; A_1_ = absorbance of ABTS + dilute solution + sample; A_2_ = absorbance of PBS + sample.

### 3.6. In Vitro Hypoglycemic Activity Assays of LJP and ULJP

The hypoglycemic activities of the LJP and ULJP in vitro were determined by α-amylase inhibitory activities following the method described in a previous study Yang et al. [55]. LJP and ULJP were previously dissolved in distilled water at different concentrations (1~8 mg/ mL). The absorbance was determined by a UV-1601 spectrophotometer (Beijing Ruili Analytical Instrument Co., Ltd., Beijin, China) against the reaction mixture without sample was used as the control. The α-amylase inhibitory activity (%) was given by [1 − (A_sample_ − A_background_)/A_control_] × 100%.

### 3.7. Effects of LJP and ULJP on RAW264.7 Macrophages

#### 3.7.1. Cell Culture

RAW264.7 macrophages were cultured in DMEM containing 10% FBS, 100 U/mL penicillin and 100 μg/mL streptomycin at 37 °C in a CO-150 incubator (New Brunswick Scientific, NJ, USA) with an atmosphere of 5% CO_2_ [56].

#### 3.7.2. Cell Viability Assay

According to the Rahmawati et al. [57] and other samples, with a slight modification, the blown cells were diluted, counted by a hemocytometer and the cell density was adjusted to 1.5 × 10^5^/mL, with 100 μL/well was seeded in a 96-well plate. To prevent edge effect, a circle of PBS solution should be added around the cells. After culturing for 24 h, the old medium was discarded, 100 μL of medium was added to the control group and 100 μL/well of LJP and ULJP medium solutions were added to the experimental group and 5 duplicate wells were set up. After adding LJP and ULJP for 24 h, add 10 μL/well of CCK-8 solution and incubate in the dark until the OD value is 0.8~1.0.

#### 3.7.3. Effects of LJP and ULJP on the Morphology of RAW264.7 Macrophages

According to published method [58,59] for the effect of the sample on the morphology of RAW264.7 macrophages, with a slight modification, RAW264.7 cells were cultured in 6-well plates with different concentrations of LJP and ULJP (100, 200, 300 μg/mL) and lipopolysaccharide (1 μg/mL) were treated for 24 h and then the morphological changes of the cells were observed.

#### 3.7.4. Effects of LJP and ULJP on Phagocytic Activity of RAW264.7 Macrophages

According to Shin et al. [58] for the effect of the sample on the phagocytic activity of RAW264.7 macrophages, with a slight modification, the neutral red uptake assay was used to measure the phagocytic activity. The cells were seeded in 96-well plates at 1 × 10^5^ cells/well and after culturing for 24 h, the old medium was removed and LJP and ULJP (100, 200, 300 μg/mL) were added respectively, incubated for 1 h and then lipopolysaccharide (1 μg/mL) and incubated for 16 h, with 4 parallel wells in each group. After the incubation, remove the old medium. In order to avoid polysaccharide or LPS residue, wash twice with PBS buffer, add 100 μL of 0.05% neutral red solution to each well and incubate for 1 h, remove the old medium, in order to avoid neutral The residual red solution was washed three times with PBS buffer and 100 μL of lysis buffer (ethanol: glacial acetic acid = 1:1) was added to each well, shaken for 60 min and its OD value was detected at a wavelength of 540 nm and RAW264.7 was calculated according to the OD value.

#### 3.7.5. Effects of LJP and ULJP on NO Release from RAW264.7 Macrophages

The standard curve test of nitrite was prepared in advance, the inflammation model was established according to 2.7.3 and the amount of NO production was measured by Griess kit [59].

#### 3.7.6. Detection of Inflammatory Factor Gene Expression by qPCR

The real-time PCR was conducted to determine iNOS mRNA. Briefly, total RNA was isolated from RAW264.7 cells using a TRIZOL reagent kit (Life technologies, Invitrogen, Carlsbad, CA, USA) according to the manufacturer’s instructions. cDNA was synthesized using the SuperScript ^®^ First-Strand synthesis system for RT-PCR (Invitrogen, Carlsbad, CA, USA) under the manufacturer’s instruction. Quantitative real-time PCR was performed on the ViiATM 7 Real-Time PCR System (Applied Biosystems, Foster city, CA, USA) with Power SYBR GREEN Master Mix (Applied Biosystems, Foster City, CA, USA). The primer sequences for iNOS and beta-actin are as follows: iNOS, 5′-CACCTTGGAGTTCACCCAGT-3′ and 5′-ACCACTCGTACTTGGGATGC-3′; beta-actin, 5′-GGACAG TGAGGCCA GG ATGG-3′ and 5′-AGTGTGACGTTGACA TCCGTAAAGA-3′.

### 3.8. Statistical Analysis

All results in this work were expressed as mean ± standard deviation of three replicates. Data in triplicate were analyzed by one-way analysis of variance using SPSS 11.5 software package for Windows (SPSS Inc., Chicago, IL, USA).

## 4. Conclusions

The results of this study prove that ultrasonic degradation is a simple and efficient method to degrade LJP and obtain ULJP. The two polysaccharides have different physicochemical properties and in vitro biological activities. Compared with LJP, ULJP has a smaller molecular weight, better antioxidant capacity, better inhibitory activity on α-amylase and stronger anti-inflammatory activity on RAW264.7 macrophages. Therefore, the ultrasonic degradation method can easily and efficiently degrade LJP and significantly improve the biological activity of LJP, which provides a theoretical basis for the in-depth utilization and research and development of *L. japonica* in the fields of medicine and food.

## Figures and Tables

**Table 1:** Chemical constituents, molecular weight parameters and monosaccharide compositions of LJP and ULJP.

Sample	LJP	ULJP
Total sugar (%)	70.10 ± 1.30	70.83 ± 1.21
Protein (%)	1.24% ± 0.02	1.15% ± 0.03
Uronic acid (%)	61.92% ± 1.01	54.95% ± 1.02
Sulfate group (%)	6.79% ± 0.23	7.78% ± 1.03
Mw (kDa)	219.678	153.895
Mn (kDa)	137.254	33.475
Mp (kDa)	181.761	7.84
Polydispersity (Mw/Mn)	1.601	4.597
Fuc	13.21	0.93
Rha	0.23	ND
Ara	0.79	0.77
Gal	9.37	72.56
Glc	0.61	16.87
Xyl	2.23	2.58
Man	4.97	0.56
Fru	ND	ND
Rib	0.17	0.35
Gal-UA	0.20	ND
Gul-UA	5.18	ND
Glc-UA	7.38	4.58
Man-UA	55.66	0.79

## Data Availability

Not applicable.
